# Nonlinear Relationship Between Coronary Perfusion Pressure and In-Hospital Outcomes After Infant Congenital Heart Surgery

**DOI:** 10.3390/children11121419

**Published:** 2024-11-24

**Authors:** Tongkai Ge, Dandong Luo, Qiuji Wang, Jimei Chen, Huanlei Huang, Chongjian Zhang

**Affiliations:** 1 Guangdong Cardiovascular Institute, Guangdong Provincial People’s Hospital, Guangdong Academy of Medical Sciences, Guangzhou 510080, China; getongkai@gdph.org.cn (T.G.); chenjimei@gdph.org.cn (J.C.); huanghuanlei@gdph.org.cn (H.H.); 2 Department of Cardiac Surgery, Guangdong Provincial People’s Hospital, Guangdong Academy of Medical Sciences, Southern Medical University, Guangzhou 510080, China

**Keywords:** coronary perfusion pressure, nonlinear, congenital heart surgery, outcomes

## Abstract

Objectives: Our goal was to evaluate the associations between postoperative coronary perfusion pressure (CPP) values and in-hospital outcomes in infants after congenital cardiac surgery. Our goal was to assess the relationship between postoperative coronary perfusion pressure (CPP) values and in-hospital outcomes in infants following congenital cardiac surgery. Methods: In this study, we conducted a retrospective analysis on a cohort of 296 consecutive infant patients (aged 31–120 days) who underwent congenital cardiac surgery between 1 January 2019 and 30 April 2019. A total of 208 patients undergoing congenital cardiac surgery were included. The primary poor in-hospital outcome was prolonged recovery. The association between CPP level and in-hospital outcomes was determined using logistic regression analysis. We also used restricted cubic splines (RCSs) to evaluate the nonlinear relationship. Results: Our study included 208 participants, among whom the mortality rate was 1%. Prolonged hospital length of stay (LOS) was defined as more than 15 days, prolonged mechanical ventilation (MV) stay as more than 96 h, and prolonged intensive care unit (ICU) LOS as more than 403 h. In univariate analyses, we found that prolonged recovery was associated with both low CPP levels (*p* < 0.001, OR 4.28, 95% CI 1.94–9.46) and high CPP levels (*p* = 0.003, OR 3.39, 95% CI 1.52–7.58). In multivariable logistic regression analysis, after full adjustment, low CPP levels and high CPP levels were significantly associated with prolonged recovery (*p* = 0.005, OR = 3.72, 95% CI 1.48–9.35 and *p* < 0.001, OR = 6.04, 95% CI 2.32–15.72, respectively). We observed that the relationship between CPP level and poor in-hospital outcomes was U-shaped in a two-piecewise linear regression analysis. We found that the inflection point of CPP level for prolonged recovery was 47 mm Hg. Conclusion: The CPP levels exhibited a nonlinear relationship with poor in-hospital outcomes.

## 1. Introduction

Congenital heart diseases affect 0.7–1% of newborns, causing significant morbidity and mortality [1]. Recent advancements in surgery have improved outcomes, but the recovery period after surgery remains delicate, which is mainly due to the varied anatomy and pathophysiology among infants [2]. The overall mortality rate of heart surgery is between 2% and 3%, while the mortality rate of some complex heart diseases exceeds 10% [3]. The occurrence of unplanned cardiac reintervention after surgery is independently associated with poor outcomes after the surgery, which also imposes considerable financial strain on families and the healthcare system [4,5]. Therefore, identifying patients who need adjustments to their treatment plans earlier is crucial.

Coronary perfusion pressure (CPP) is the pressure exerted on the coronary artery blood supply during the cardiac cycle, and it is essential for maintaining normal heart muscle function and determining myocardial tissue perfusion [6]. CPP is widely recognized as a crucial indicator for assessing the effectiveness of cardiopulmonary resuscitation [7]. Studies have proved that a sufficient CPP level is necessary for survival after cardiac arrest [8]. In addition, CPP is often defined as the disparity between diastolic blood pressure (DBP) and central venous pressure (CVP) [7]. DBP level and CVP have been proven to have an association with clinical outcomes in cardiovascular disease. A low DBP is correlated with subclinical myocardial ischemia and other poor outcomes, while elevated CVP is positively associated with postoperative complications after cardiac surgery [9,10,11]. As a combination of DBP and CVP, CPP has been demonstrated to be a predictor of mortality in patients presenting with cardiogenic shock [12]. Mazimba et al. found that a CPP level below 40 mmHg was associated with adverse outcomes in adult patients with advanced heart failure [13]. In addition, Buchanan et al. reported that a low CPP level was positively associated with worse in-hospital outcomes and increased the risk of cardiovascular collapse in patients following percutaneous coronary intervention [14]. From a clinical perspective, CPP is an indicator that is easy to measure. By monitoring and adjusting the CPP, doctors can identify patients with hemodynamic instability early and take appropriate treatment measures to prevent adverse outcomes. However, there is limited research on the relationships between the CPP level and outcomes in infant patients undergoing congenital cardiac surgery. We have observed that both excessively high and low CPP levels are often linked to poor outcomes in infants after congenital cardiac surgery. Thus, we hypothesized that the CPP level has a nonlinear relationship with in-hospital outcomes after congenital cardiac surgery. We aim to assess the associations and analyze the impact of postoperative CPP values on in-hospital outcomes in infants after congenital cardiac surgery.

## 2. Materials and Methods

### 2.1. Study Population and Design

In this study, we conducted a retrospective analysis of a cohort of 296 consecutive infant patients (aged 31–120 days) who underwent congenital cardiac surgery between 1 January 2019 and 30 April 2019. We excluded patients who were aged under 30 days (*n* = 25), underwent surgery without cardiopulmonary bypass (*n* = 30), required mechanical ventilation before surgery (*n* = 31), and required extracorporeal membrane oxygenation (ECMO) after surgery (*n* = 7). In total, 208 patients undergoing congenital cardiac surgery were included. The population was categorized into tertiles based on CPP levels (high-tertile group, moderate-tertile group, and low-tertile group).

This retrospective study was conducted in compliance with the principles outlined in the 1964 Helsinki Declaration, and it received approval from the Research Ethics Committee at Guangdong Provincial People’s Hospital. Informed consent was obtained for this study (KY2024-150-01).

### 2.2. Clinical Data

We extracted demographic, clinical, laboratory, and surgical details from electronic medical records. Preoperative blood chemistry measurements, including hemoglobin levels, platelet count, ALT levels, and creatinine levels, were acquired. Intraoperative clinical data, such as the time of cardiopulmonary bypass, aortic cross-clamping, and blood transfusion, were extracted from the surgical records. In our center, we use the Risk Adjustment for Congenital Heart Surgery 1 (RACHS-1) to categorize the complexity of cardiac surgical procedures. We believe that it provides a more comprehensive and accurate representation of all categories of congenital heart diseases. The pediatric cardiac surgery intensive care unit (ICU) at our center is tailored to patients following cardiac surgery, with a skilled team of physicians and nurses experienced in managing the unique needs of infants. After cardiac surgery, the functions of organs must be closely monitored to allow sufficient time for improving cardiac function. Doctors and nurses must monitor physiological data at all times and make urgent clinical decisions promptly. Initially, most children require a nurse-to-patient ratio of 1:1. Once the child’s condition stabilizes, especially when mechanical ventilation is no longer required, a nurse-to-patient ratio of 2:1 can be considered.

We extracted all available postoperative data elements, including heart rate (HR), SBP, DBP, CVP, and vasoactive inotrope score (VIS) from the nursing record charts. The recorded time point was at the first hour after admission to the cardiac surgery ICU (SICU).

The following manners were used to calculate the CPP and VIS: CPP [7] = DBP − CVP VIS [15] = dopamine dose (micrograms/kilogram/min) + dobutamine dose (micrograms/kilogram/min) + 100 × epinephrine (micrograms/kilogram/min) + 10 × milrinonedose (micrograms/kilogram/min) + 10,000 × asopressin dose (U/kilogram/min) + 100 × norepinephrine dose (micrograms/kilogram/min).

Patients in the upper quartile of the length of stay (LOS) in the hospital were defined as having a prolonged hospital length of stay (LOS). Patients in the upper quartile of mechanical ventilation stay were defined as having prolonged mechanical ventilation (MV). Patients in the upper quartile the length of stay (LOS) in the ICU were defined as having a prolonged ICU length of stay (LOS).

The primary poor in-hospital outcome was a composite outcome (prolonged recovery) that included any patient with prolonged LOS, prolonged MV, or prolonged ICU LOS. We chose to evaluate the outcomes using tertile cut-points of the CPP level for the entire cohort.

### 2.3. Statistical Analysis

The CPP level was stratified into three groups: the high-tertile group, the moderate-tertile group, and the low-tertile group. Continuous data are presented as medians (interquartile range) or means ± SD, and descriptive data are reported as frequencies. The distributions of continuous data were tested for normality using the Kolmogorov–Smirnov test. Statistical differences among groups of continuous variables following a normal distribution were assessed using a one-way analysis of variance, while the Kruskal-Wallis test was used for data with a skewed distribution. The chi-square test was used to compare categorical variables, which are reported as percentages. The association between the CPP level and in-hospital outcomes was determined using logistic regression, and the results are expressed as the odds ratio (OR) and 95% confidence intervals (CIs). Table 1 presents the results of the unadjusted model, minimally adjusted model, and fully adjusted model analyses. In the minimally adjusted model, the covariates were adjusted for height, weight, male gender, age, gestation, preoperative hemoglobin, preoperative platelet count, preoperative ALT, and preoperative creatinine; in the fully adjusted model, the covariates were adjusted for height, weight, male gender, age, gestation, preoperative hemoglobin, preoperative platelet count, preoperative ALT, preoperative creatinine, RACHS-1, transfusion, cardiopulmonary bypass time, aortic cross-clamping time, heart rate, and VIS. We used a restricted cubic spline (RCS) regression model to evaluate the nonlinear relationship between the CPP level and in-hospital outcomes. The inflection point or turning point was calculated using a two-piecewise linear regression model. We used a log-likelihood ratio test to compare a one-line linear regression model (model I) with the two-piecewise linear model (model II). The statistical analyses were conducted using the statistical software R-4.2.2. A *p*-value of *p* < 0.05 was considered statistically significant.

## 3. Results

### 3.1. Baseline Patient Characteristics

Our study included 208 participants. Table 2 describes the baseline characteristics and compares the laboratory parameters, scoring systems, and outcomes. A summary of the primary diagnoses of the population is provided in Supplementary Table SE. The median hospital length of stay was 9.3 (6.1, 15.2) days. The median duration of mechanical ventilation was 26 (8, 96) hours, and the median length of stay in the ICU was 229 (67.5, 403) hours. A prolonged hospital LOS was defined as exceeding 15 days. Prolonged MV was defined as lasting more than 96 hours, and a prolonged ICU LOS was defined as exceeding 403 hours.

The median CPP level was 51 (48, 58.3) mmHg. The minimum CPP level was 19 mmHg, and the maximum CPP level was 83 mmHg. The study population’s clinical characteristics were categorized into three groups based on the tertiles of the CPP levels: the low-tertile group (19–45 mmHg), the moderate-tertile group (45–55 mmHg), and the high-tertile group (56–83 mmHg). There were no differences in gender, age, weight, height, gestation, preoperative hemoglobin, preoperative ALT, preoperative creatinine, transfusion of RBCs, cardiopulmonary bypass time, aortic cross-clamping time, vasoactive inotrope score, or mortality rate among the three groups. The RACHS-1 score, heart rate, systolic blood pressure, coronary perfusion pressure, diastolic blood pressure, ICU LOS, hospital LOS, mechanical ventilation time, rate of prolonged recovery, prolonged ICU LOS, and prolonged MV were significantly different among the three groups (Table 2).

### 3.2. Univariate Analyses of the Associations Between Risk Factors and Poor In-Hospital Outcomes

To identify which variables affected poor in-hospital outcomes, univariate analyses were conducted. As illustrated in Table 3, we found that prolonged recovery was associated with preoperative creatinine (*p* = 0.001, OR = 1.08, 95% CI 1.03–1.13), postoperative heart rate (*p* = 0.007, OR = 1.02, 95% CI 1.01–1.03), systolic blood pressure (*p* = 0.001, OR = 0.98, 95% CI 0.96–0.99), RACHS-1 category 3 (*p* = 0.01, OR = 5.15, 95% CI 1.44–18.36), low CPP levels (*p* < 0.001, OR = 4.28, 95% CI 1.94–9.46), and high CPP levels (*p* = 0.003, OR = 3.39, 95% CI 1.52–7.58).

Moreover, we found that prolonged hospital LOS was associated with preoperative creatinine (*p* = 0.009, OR = 1.05, 95% CI 1.01–1.09), postoperative heart rate (*p* = 0.007, OR = 1.02, 95% CI 1.01–1.04), RACHS-1 category 3 (*p* = 0.03, OR = 4.95, 95% CI 1.17–21.03), low CPP levels (*p* < 0.01, OR = 5.88, 95% CI 2.25–15.39), and high CPP levels (*p* = 0.035, OR = 2.94, 95% CI 1.08–8.01). Prolonged MV was associated with age (*p* = 0.025, OR = 1.00, 95% CI 0.99–1.00), preoperative creatinine (*p* < 0.001, OR = 1.08, 95% CI 1.03–1.13), postoperative heart rate (*p* = 0.009, OR = 1.02, 95% CI 1.01–1.04), RACHS-1 category 3 (*p* = 0.005, OR = 10.62, 95% CI 2.04–55.33), low CPP levels (*p* = 0.002, OR = 4.17, 95% CI 1.66–10.45), and high CPP levels (*p* = 0.043, OR = 2.67, 95% CI 1.03–6.92) (Supplementary Table S1). Additionally, prolonged ICU LOS was linked with preoperative creatinine (*p* = 0.003, OR = 1.06, 95% CI 1.02–1.11), postoperative heart rate (*p* = 0.008, OR = 1.02, 95% CI 1.01–1.04), low CPP levels (*p* < 0.001, OR = 7.6, 95% CI 2.73–21.17), and high CPP levels (*p* = 0.019, OR = 3.59, 95% CI 1.24–10.40) (Supplementary Table S2).

### 3.3. Association of the Cpp Level with In-Hospital Outcomes

**Figure 1:**
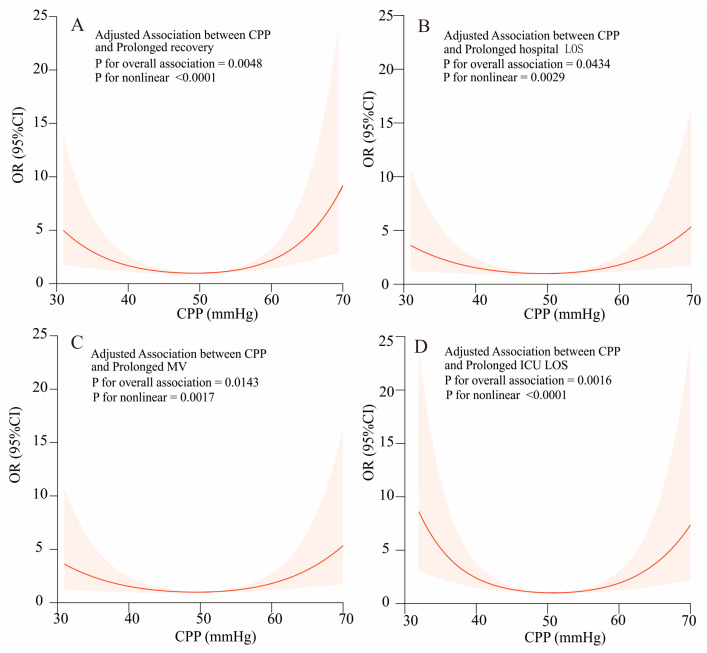
The restricted cubic spline (RCS) regression analysis between the CPP level and poor in-hospital outcomes. The graph displays only the data within the 95% confidence interval, so the range 30–70 mmHg is plotted on the *X*-axis.

When CPP was modeled in the standard linear regression (Model I), the CPP level one hour after cardiac surgery had no significant association with in-hospital poor outcomes (prolonged recovery (*p* = 0.28), prolonged hospital LOS (*p* = 0.61), prolonged MV (*p* = 0.45), and prolonged ICU LOS (*p* = 0.52)). Conversely, the nonlinear multivariable model was well calibrated (Table 4). We observed a nonlinear association between the CPP level and prolonged recovery (Figure 1A).

As shown on the left side, when the CPP level was less than 47 mmHg, the incidence of prolonged recovery decreased by 13% for each unit decrease (OR = 0.87, 95% CI 0.81–0.94; *p* < 0.001). As shown on the right side, when the CPP level was more than 47 mmHg, the incidence of prolonged hospital stay increased by 13% for each unit increase (OR = 1.13, 95% CI 1.07–1.20; *p* < 0.001).

Similarly, Figure 1B highlights the nonlinear relationship between the CPP level and prolonged hospital LOS, with an inflection point at 52 mmHg (log-likelihood ratio test, *p* < 0.001, Table 4). Additionally, Figure 1C depicts the nonlinear relationship between the CPP level and prolonged MV (*p* for nonlinearity = 0.002), with an inflection point at 50 mmHg (Table 4). Figure 1D illustrates the nonlinear relationship between the CPP level and prolonged ICU LOS (Table 4), with an inflection point at 48 mmHg (Table 4).

In the sensitivity analysis, we classified the CPP level and selected the moderate CPP level group as the reference group for comparison. In the multivariable logistic regression analysis, low CPP levels and high CPP levels were significantly associated with prolonged recovery (*p* < 0.001, OR = 4.28, 95% CI 1.94–9.46 and *p* = 0.003, OR = 3.39, 95% CI 1.52–7.58, respectively). After adjusting for height, weight, male gender, age, gestation, preoperative hemoglobin, preoperative platelet count, preoperative ALT, and preoperative creatinine, the association remained significant (*p* = 0.002, OR = 3.66, 95% CI 1.59–8.42 and *p* = 0.002, OR = 4.00, 95% CI 1.70–9.37, respectively). Following further adjustments for height, weight, male gender, age, gestation, preoperative hemoglobin, preoperative platelet count, preoperative ALT, preoperative creatinine, RACHS-1, transfusion, cardiopulmonary bypass time, aortic cross-clamping time, heart rate, and VIS revealed that both low and high CPP levels were found to be significantly associated with prolonged recovery (*p* = 0.005, OR = 3.72, 95% CI 1.48–9.35 and *p* < 0.001, OR = 6.04, 95% CI 2.32–15.72, respectively). In the unadjusted model, minimally adjusted model, and fully adjusted model, low CPP levels and high CPP levels were also significantly associated with prolonged hospital LOS, prolonged MV, and prolonged ICU LOS (Table 4).

## 4. Discussion

In our retrospective cohort study, we found that the relationship between CPP levels and poor in-hospital outcomes after congenital cardiac surgery in infants was U-shaped. We also found that the inflection point for prolonged recovery was 47 mmHg (Figure 1A, Table 4).

Many institutions have aimed to improve postoperative outcomes following congenital heart surgery by concentrating on minimizing mortality and complications. Scoring indices are frequently used as tools for the early detection of adverse outcomes. For instance, the Risk Adjustment for Congenital Heart Surgery (RACHS-1) method and the Society of Thoracic Surgeons European Association for Cardio-Thoracic Surgery (STS-EACTS) mortality score are widely accepted, and they are primarily focused on mortality [16,17]. The vasoactive-ventilation-renal score and the vasoactive inotropic score, calculated within 24 h after cardiac surgery, were strongly associated with poor in-hospital outcomes [2,18]. Early hemodynamic disturbances are also recognized to be correlated with adverse outcomes in a variety of clinical scenarios [19]. Our results suggest that adjusting CPP levels for infant patients after congenital cardiac surgery could be a simple and effective tool for improving in-hospital outcomes.

Previous studies have found correlations between CPP levels and outcomes in various disease contexts. In patients or models experiencing ventricular fibrillation cardiac arrest and undergoing cardiopulmonary resuscitation, a CPP level of >20 mmHg is associated with both acute resuscitative success and survival [8,20]. When the CPP level was <42 mmHg, patients with left ventricular systolic dysfunction undergoing reasonable incomplete revascularization had a significantly higher risk of mortality than that of those undergoing complete revascularization [21]. Similarly, a CPP below 40 mmHg in advanced heart failure patients undergoing invasive hemodynamic monitoring was linked to adverse outcomes [22]. Hagel et al. [23] found that a CPP of <35 mm Hg was associated with adverse outcomes after cardiac surgery in children. These findings suggested that the CPP level was negatively linked with adverse outcomes in a linear relationship. Consistently, our study observed that a level of <47 mm Hg was negatively associated with adverse outcomes.

However, this linear relationship may be problematic. Dr. Böhm et al. [24] suggested that DBP potentially influences CPP, showing an inverted J-shaped relationship with adverse clinical outcomes. DBP plays a crucial role in myocardial perfusion, as 85% of left ventricular perfusion occurs during the diastolic phase. When the DBP is low, it results in a decrease in effective CPP and leads to increased filling pressures. Consequently, this further reduces the perfusion gradient. It is well known that higher blood pressure is linked to higher mortality and poor outcomes. In patients with longstanding hypertension, the range of coronary arterial autoregulation is diminished, particularly in the subendocardium. This reduction can result in decreased CPP and subsequently lead to subendocardial ischemia. However, this study involved CPP but did not analyze the relationship between CPP and outcomes. Interestingly, Hamud et al. [25] investigated the potential nonlinear association between right coronary artery perfusion pressure (RCDPP) and mortality in an inverted J-shaped curve. This study explained the variability in RCDPP mainly through changes in DBP. In addition, categorizing the CPP level while neglecting the potential nonlinear impact on patient outcomes reduces statistical power. In our study, when the CPP was analyzed without considering nonlinear relationships, the median CPP level during the first hour after cardiac surgery did not show a significant association with poor in-hospital outcomes. On the contrary, when the nonlinear model was properly calibrated, we found that the CPP level measured within the first hour postoperatively could be accurately linked to outcomes in infants following cardiac surgery.

To our knowledge, this is the first study to discover the nonlinear association between the CPP level and adverse in-hospital outcomes in infants after cardiac surgery. Our hypothesis and study results are indeed in line with the evidence indicating that both excessively low and excessively high CPP levels may worsen outcomes in infants undergoing cardiac surgery. This phenomenon mirrors the nonlinear impact of certain physiological imbalances on patient outcomes [26,27]. The shape of the curve can vary—a J-curve, U-curve, or reverse J-curve—depending on the location of the inflection point [28]. Our study found a U-shaped association between the CPP level and adverse outcomes, but the underlying mechanisms are not well known. The negative association between the CPP level and adverse outcomes can be explained by myocardial blood flow. When the CPP level is between 40 and 120 mmHg in animal models, the coronary artery regulates the expansion and contraction of microvessels to adjust vascular resistance, ensuring stable coronary blood flow [29]. An increase in coronary blood flow directly corresponds to a linear increase in CPP, which affects myocardial function. If the CPP level is below a certain threshold, this indicates that coronary blood flow is insufficient to maintain myocardial perfusion [30]. However, few studies have reported a positive association between the CPP level and adverse outcomes. In isovolumetrically working guinea pig hearts, raising the CPP level from 60 to 80 mmHg increased oxygen consumption by 32.7 ± 6.2% [31]. Bai et al. [32] reported that a high CPP level significantly increased oxygen consumption, especially when autoregulation was poor. With the U-shaped association between the CPP level and adverse outcomes, we speculated that the variability in CPP is mainly driven by changes in DBP. The CPP level can be a double-edged sword, as both excessively low and excessively high levels have adverse effects. Therefore, in clinical practice, abnormal CPP can serve as a warning sign indicating an increased risk of adverse events. This information can prompt physicians to make necessary adjustments to medications or therapeutic approaches.

One major limitation of our study was the reliance on the value at a single time point for each variable to characterize hemodynamics. To address this issue, future studies should consider analyzing CPP levels at multiple time points rather than a single time point. Additionally, in this study, we observed that the relationship between CPP levels and poor in-hospital out-comes was U-shaped after congenital cardiac surgery in infants. This is different from the linear relationship reported in previous studies. Baseline CPP, at the level of <47 mm Hg, was negatively associated with adverse outcomes. When the CPP level reached > 47 mmHg, the CPP level was positively associated with adverse outcomes. However, we chose to assess the outcomes using tertile cutoff points of the CPP level for the entire cohort. In future, we can employ ROC analysis to find the low and high cutoff CPP values for predicting adverse results for each studied outcome, which will offer more beneficial guidance for clinical practice.

## 5. Conclusions

In conclusion, we found that CPP levels exhibited a nonlinear relationship with adverse outcomes, with a U-shaped pattern. CPP levels that were too low and too high were associated with adverse outcomes.

## Figures and Tables

**Table 1:** Multivariate analysis of the CPP level with poor in-hospital outcomes.

	Unadjusted Model OR (95%CI) *p*	Minimally Adjusted Model OR (95%CI) *p*	Fully Adjusted Model OR (95%CI) *p*
Prolonged recovery			
CPP	0.99 (0.97–1.01, *p* = 0.290)	0.99 (0.97–1.01, *p* = 0.289)	0.99 (0.97–1.01, *p* = 0.290)
CPP (tertiles)			
moderate	1(ref)	1(ref)	1(ref)
low	4.28 (1.94–9.46, *p* < 0.001)	3.66 (1.59–8.42, *p* = 0.002)	3.72 (1.48–9.35, *p* = 0.005)
high	3.39 (1.52–7.58, *p* = 0.003)	4.00 (1.70–9.37, *p* = 0.002)	6.04 (2.32–15.72, *p* < 0.001)
Prolonged MV			
CPP	0.98 (0.95–1.01) *p* = 0.117	1.00 (0.97–1.03) *p* = 0.801	1.01 (0.98–1.04) *p* = 0.610
CPP (tertiles)			
moderate	1(ref)	1(ref)	1(ref)
low	4.17 (1.66–10.45) *p* = 0.002	3.44 (1.29–9.12) *p* = 0.013	3.77 (1.26–11.32) *p* = 0.018
high	2.67 (1.03–6.91) *p* = 0.043	3.39 (1.24–9.28) *p* = 0.018	5.34 (1.73–16.49) *p* = 0.004
Prolonged hospital LOS			
CPP	0.97 (0.94–1.00) *p* = 0.025	0.98 (0.95–1.00) *p* = 0.094	0.98 (0.95–1.01) *p* = 0.259
CPP (tertiles)			
moderate	1(ref)	1(ref)	1(ref)
low	5.88 (2.25–15.39) *p* < 0.001	5.20 (1.94–13.89) *p* = 0.001	3.77 (1.26–11.32) *p* = 0.018
high	2.94 (1.08–8.01) *p* = 0.035	2.98 (1.07–8.30) *p* = 0.037	5.34 (1.73–16.49) *p* = 0.004
Prolonged ICU LOS			
CPP	0.97 (0.94–0.99) *p* = 0.013	0.98 (0.95–1.00) *p* = 0.100	0.98 (0.95–1.02) *p* = 0.342
CPP (tertiles)			
moderate	1(ref)	1(ref)	1(ref)
low	7.60 (2.73–21.17) *p* < 0.001	6.76 (2.35–19.41) *p* < 0.001	7.25 (2.28–23.04) *p* < 0.001
high	3.59 (1.24–10.40) *p* = 0.019	4.08 (1.36–12.23) *p* = 0.012	5.68 (1.67–19.29) *p* = 0.005

LOS, length of stay; MV, mechanical ventilation; OR, odds ratio; minimally adjusted model: adjusted for height, weight, male gender, age, gestation, preoperative hemoglobin, preoperative platelet count, preoperative ALT, and preoperative creatinine; fully adjusted model: adjusted for height, weight, male gender, age, gestation, preoperative hemoglobin, preoperative platelet count, preoperative ALT, preoperative creatinine, RACHS_1, transfusion, cardiopulmonary bypass time, aortic cross-clamping time, heart rate, and VIS.

**Table 2:** Baseline characteristics and outcomes.

CPP Groups	Low Tertile	Moderate Tertile	High Tertile	*p*
	(19~45 mmHg)	(45~55 mmHg)	(56~83 mmHg)	
**Baseline characteristics**				
N	75	62	71	
Male (%)	39 (52.0%)	35 (56.5%)	42 (59.2%)	0.679
Age (day)	151.0 (78.5;227.5)	148.5 (75.0;209.0)	185.0 (119.5;230.5)	0.226
Height (cm)	61.0 (55.5;66.5)	60.5(55.0;67.0)	63.0 (58.0;67.5)	0.241
Weight (kg)	5.3 (4.3; 7.0)	5.5 (4.5; 6.5)	6.5 (5.0; 7.5)	0.059
Gestation (weeks)	40.0 (38.0;40.0)	40.0 (38.0;40.0)	39.0 (38.0;40.0)	0.410
Preoperative hemoglobin (g/L)	114.0 (105.0;127.5)	112.5 (105.0;124.0)	109.0 (103.5;118.5)	0.190
Preoperative platelet(10^9^/L)	357.0 (304.5;456.5)	377.0 (316.0;452.0)	374.0 (323.0;456.5)	0.531
Preoperative ALT (U/L)	22.0 (16.0;30.5)	27.0 (20.0;34.0)	24.0 (18.0;31.0)	0.083
Preoperative creatinine (µmol/L)	18.9 (15.6;24.3)	17.1 (15.0;22.6)	17.7 (15.0;21.2)	0.071
RACHS-1 score(%)				0.014
1	6 (8.0%)	7 (11.3%)	6 (8.5%)	
2	49 (65.3%)	47 (75.8%)	62 (87.3%)	
3	18 (24.0%)	7 (11.3%)	2 (2.8%)	
4	2 ( 2.7%)	1 (1.6%)	1 (1.4%)	
Transfusion of RBCs (U)	2.0 (1.0; 2.8)	2.5 (1.5; 3.0)	2.0 (1.0; 3.0)	0.14
Cardiopulmonary bypass time (min)	70.0 (60.5;94.5)	77.0 (58.0;89.0)	73.0 (58.5;101.0)	0.729
Aortic cross clamp time (min)	40.0 (31.0;55.5)	43.5 (32.0;54.0)	40.0 (29.0;60.0)	0.793
Heart rate (beats/min)	144.1 ± 19.3	132.0 ± 19.2	132.5 ± 21.5	<0.001
Systolic blood pressure (mmHg)	76.8 ± 15.7	96.6 ± 12.5	110.7 ± 13.1	<0.001
Central venous pressure (mmHg)	6.0 (5.0; 8.0)	6.0 (5.0; 8.0)	6.0 (4.0; 7.0)	0.024
Coronary perfusion pressure (mmHg)	36.0 (33.0;41.0)	51.0 (48.0;53.0)	62.0 (58.0;67.0)	<0.001
Vasoactive-inotrope score	5 (3.0;5.2)	5 (3.0;8.0)	5 (0;8.0)	0.85
Diastolic blood pressure (mmHg)	43.1 ± 5.8	57.1 ± 4.0	69.0 ± 5.9	<0.001
**Outcomes**				
ICU LOS (hours)	290.0 (81.5;540.5)	134.0 (51.0;259.0)	236.0 (94.5;398.5)	0.001
Hospital LOS (days)	12.1 (7.0;17.1)	7.1 (5.9; 9.2)	10.1 (6.8;14.8)	<0.001
Mechanical ventilation (hours) Prolonged recovery n (%)	70.0 (13.0;140.5) 36 (48.0%)	16.5 (6.0;52.0) 11 (17.7.4%)	23.0 (7.5;97.5) 30 (42.3%)	<0.001 0.001
Prolonged hospital LOS n (%)	29 (38.7%)	6 (9.7%)	17 (23.9%)	<0.001
Prolonged MV n (%)	26 (34.7%)	7 (11.3%)	18 (25.4%)	0.007
Prolonged ICU LOS n (%)	30 (40.0%)	5 (8.1%)	18 (25.4%)	<0.001
Mortality (%) n (%)	1(1.3%)	1 (1.6%)	0 (0.0%)	0.584

ALT, alanine aminotransferase; RACHS = Risk Adjustment for Congenital Heart Surgery; min, minute; RBC, red blood cell; MV, mechanical ventilation; LOS, length of stay.

**Table 3:** Univariate analyses of associations between risk factors and prolonged recovery.

Name	Univariable Analyses
	Prolonged Recovery
Variables	(OR, 95%CI, *p*)
CPP groups	
Moderate	1(ref)
Low	4.28 (1.94–9.46) *p* < 0.001
High	3.39 (1.52–7.58) *p* = 0.003
Height (cm)	0.98 (0.94–1.02) *p* = 0.36
Weight (kg)	0.91 (0.77–1.07) *p* = 0.24
Male (%)	0.85 (0.48–1.50) *p* = 0.57
Age (days)	1.00 (0.99–1.00) *p* = 0.21
Gestation (weeks)	0.97 (0.86–1.09) *p* = 0.60
RACHS_1 score	
1	1(ref)
2	0.97 (0.35–2.71) *p* = 0.96
3	5.15 (1.44–18.36) p = 0.01
4	6.50 (0.55–76.18) *p* = 0.14
Transfusion of RBC (u)	1.14 (0.96–1.36) *p* = 0.13
Cardiopulmonary bypass time (min)	1.00 (0.99–1.00) *p* = 0.34
Aortic cross clamp time (min)	0.99 (0.98–1.00) *p* = 0.19
Preoperative hemoglobin (g/L)	1.01 (0.99–1.02) *p* = 0.51
Preoperative platelet (10^9^/L)	1.00 (1.00–1.00) *p* = 0.56
Preoperative ALT (U/L)	0.99 (0.97–1.00) *p* = 0.11
Preoperative creatinine (µmol/L)	1.08 (1.03–1.13) *p* = 0.001
Heart rate (beats/min)	1.02 (1.01–1.03) *p* = 0.007
Central venous pressure (mmHg) Systolic blood pressure (mmHg) Diastolic blood pressure (mmHg) Vasoactive inotrope score	1.01 (0.90–1.13) *p* = 0.84 0.98 (0.96–0.99) *p* = 0.001 0.99 (0.96–1.01) *p* = 0.29 1.01 (0.99–1.03) *p* = 0.44

CPP, coronary perfusion pressure; min, minute; RACHS = Risk Adjustment for Congenital Heart Surgery; RBC, red blood cell; ALT, alanine aminotransferase;

**Table 4:** Threshold effect analysis of CPP with respect to outcomes using two-piecewise linear regression.

	Prolonged Recovery	Prolonged Hospital LOS	Prolonged MV	Prolonged ICU LOS
	OR (95%CI) *p*	OR (95%CI) *p*	OR (95%CI) *p*	OR (95%CI) *p*
Model I	1.02 (0.99,1.05) 0.28	0.99 (0.96, 1.02) 0.61	1.01 (0.98, 1.05) 0.45	0.99 (0.96, 1.02) 0.52
Model II				
Turning point (K)	47	52	50	48
<K effect 1	0.87(0.81,0.94) < 0.001	0.92 (0.87, 0.97) 0.002	0.92 (0.87, 0.99) 0.015	0.84 (0.78, 0.91) < 0.001
>K effect 2	1.13(1.07,1.20) <0.001	1.10 (1.03, 1.17) 0.004	1.11 (1.04, 1.19) 0.001	1.13 (1.06, 1.21) <0.001
*p* for log-likelihood ratio test	<0.001	<0.001	<0.001	<0.001

Model I, standard linear regression; Model II, two-piecewise linear regression; LOS, length of stay; MV, mechanical ventilation; OR, odds ratio.

## Data Availability

Data will be made available upon request.

## Supplementary Materials

The following supporting information can be downloaded at: https://www.mdpi.com/article/10.3390/children11121419/s1. Supplementary Table S1: Univariate analyses of risk factors associated with the prolonged MV; Supplementary Table S2: Univariate analyses of risk factors associated with the prolonged ICU LOS; Table SE: Primary diagnosis of the population.
