# Supplementary material for: Nonlinear Relationship Between Coronary Perfusion Pressure and In-Hospital Outcomes After Infant Congenital Heart Surgery
Source: Children (Basel). 2024 Nov 24;11(12):1419. doi: 10.3390/children11121419 (PMC11674232; doi:10.3390/children11121419)
Supplement: Supplementary file 1 [file children-11-01419-s001.zip › children-3214980-supplementary.pdf]

Supplementary Table S1 Univariate Analyses of Risk Factors Associated with the prolonged MV

**Name**		**Univariable analyses**
		**Prolonged MV**
**Variables**		**(OR, 95%CI, P)**
CPP groups		
moderate		(1)ref
low		4.17 (1.66-10.45, p=0.002)
high		2.67 (1.03-6.91, p=0.043)
Height(cm)		0.97 (0.92-1.01, p=0.132)
Weight(kg)		0.85 (0.70-1.03, p=0.096)
Male (%)		1.06 (0.56-2.01, p=0.856)
Age(days)		1.00 (0.99-1.00, p=0.025)
Gestation(weeks)		0.97 (0.85-1.11, p=0.676)
RACHS_1 score (%)		
	1	ref
	2	2.16 (0.47-9.83, p=0.320)
	3	10.62(2.04-55.33, p=0.005)
	4	8.50 (0.74-98.21, p=0.086)
transfusion of RBC(u)		1.20 (1.00-1.45, p=0.052)
Cardiopulmonary bypass time(min)		1.00 (0.99-1.01, p=0.404)
Aortic cross clamp time(min)		0.99 (0.98-1.01, p=0.309)
Preoperative hemoglobin(g/L)		1.01 (1.00-1.03, p=0.154)
Preoperative platelet(10^9/L)		1.00 (1.00-1.00, p=0.197)
Preoperative ALT (U/L)		0.99 (0.97-1.01, p=0.386)
Preoperative creatinine(μmol/L)		1.08 (1.03-1.13, p<0.001)
Heart rate(beats/min)		1.02 (1.01-1.04, p=0.009)
Central venous pressure(mmHg)		1.07 (0.94-1.21, p=0.301)

CPP, coronary perfusion pressure; MV, Mechanical ventilation; min, minute;

RACHS_1, Risk Adjustment for Congenital Heart Surgery 1; RBC, red blood cell; ALT, alanine aminotransferase;

Supplementary Table S2 Univariate Analyses of Risk Factors Associated with the prolonged ICU LOS

Name	Univariable analyses
	**Prolonged ICU LOS**
**Variables**	**(OR, 95%CI, P)**
CPP groups	
moderate	(1)ref
low	7.60 (2.73-21.17, p<0.001)
high	3.59 (1.24-10.40, p=0.019)
Height(cm)	0.96 (0.92-1.01, p=0.100)
Weight(kg)	0.84 (0.69-1.01, p=0.068)
Male (%)	
	0.81 (0.43-1.53, p=0.519)
Age(days)	1.00 (0.99-1.00, p=0.105)
Gestation(weeks)	0.91 (0.80-1.04, p=0.184)
RACHS_1 score(%)	
1	ref
2	0.63 (0.21-1.89, p=0.408)
3	3.50 (0.98-12.49, p=0.054)
4	8.40 (0.70-100.59, p=0.093)
transfusion of RBC(u)	1.17 (0.98-1.41, p=0.088)
Cardiopulmonary bypass time(min)	1.00 (0.99-1.01, p=0.522)
Aortic cross clamp time(min)	0.99 (0.98-1.01, p=0.212)
Preoperative hemoglobin(g/L)	1.01 (0.99-1.03, p=0.373)
Preoperative platelet(109/L)	1.00 (1.00-1.00, p=0.246)
Preoperative ALT (U/L)	0.99 (0.97-1.01, p=0.426)
Preoperative creatinine(μmol/L)	1.06 (1.02-1.11, p=0.003)
Heart rate(beats/min)	1.02 (1.01-1.04, p=0.008)
Central venous pressure(mmHg)	0.99 (0.87-1.12, p=0.885)

CPP, coronary perfusion pressure; RACHS_1, Risk Adjustment for Congenital Heart Surgery; RBC, red blood cell; min, minute; ALT, alanine aminotransferase;

Table SE Primary diagnosis of the population

**Diagnosis**	**Count(n)**<br>**All (208)**
APW	1
APW, ASD, MR	1
ASD	4
ASD, MR	1
ASD, MR, PDA	1
ASD, PDA	1
ASD, VSD	2

ASD, VSD, PDA	7
ASD, VSD, MR, PDA	1
VSD	30
VSD, PFO	40
VSD, PDA	15
VSD, PFO, PDA	24
AVSD	2
AVSD, PR	2
COA, VSD, ASD	4
COA, VSD, PDA	5
PS, ASD	11
PS, ASD, VSD	8
PS, VSD, PDA	4
TAPVC, ASD	6
TGA, VSD	2
TGA, PS, VSD, ASD	1
TGA, VSD, ASD	3
TOF	10
TOF, PDA	7
ALCAPA	3
PDA, MR	8
DORV	1
DORV, PS	1
SV, Ebstein	1
SV, COA	1

APW, aorta-pulmonary window; ASD, atrial septal defect; PFO, patent foramen ovale; MR, mitral valve regurgitation; PDA, patent ductus arteriosus; VSD, ventricular septal defect; AVSD, atrioventricular septal defect; COA, coarctation of the aorta; PS, pulmonary artery stenosis; TAPVC, total anomalous pulmonary venous connection; TGA, transposition of the great arteries; TOF, tetralogy of fallot; ALCAPA, anormalous origin of left coronary artery from the pulmonary Artery; DORV, double-outlet right ventricle; SV, single ventricle; PR, Pulmonary artery regurgitation
